# Artificially Induced Pluripotent Stem Cell-Derived Whole-Brain Organoid for Modelling the Pathophysiology of Metachromatic Leukodystrophy and Drug Repurposing

**DOI:** 10.3390/biomedicines9040440

**Published:** 2021-04-20

**Authors:** Sally Esmail, Wayne R. Danter

**Affiliations:** 123 Genetix, 11280 Riverside Drive E, Windsor, ON N8P 1A4, Canada; sesmai2@uwo.ca

**Keywords:** metachromatic leukodystrophy, artificially induced whole-brain organoids, drug repurposing, drug discovery, rare metabolic disorder

## Abstract

Metachromatic leukodystrophy (MLD) is a rare neurodegenerative disease that results from a deficiency of the lysosomal enzyme arylsulfatase A (ARSA). Worldwide, there are between one in 40,000 and one in 160,000 people living with the disease. While there are currently no effective treatments for MLD, induced pluripotent stem cell-derived brain organoids have the potential to provide a better understanding of MLD pathogenesis. However, developing brain organoid models is expensive, time consuming and may not accurately reflect disease progression. Using accurate and inexpensive computer simulations of human brain organoids could overcome the current limitations. Artificially induced whole-brain organoids (aiWBO) have the potential to greatly expand our ability to model MLD and guide future wet lab research. In this study, we have upgraded and validated our artificially induced whole-brain organoid platform (NEUBOrg) using our previously validated machine learning platform, DeepNEU (v6.2). Using this upgraded NEUBorg, we have generated aiWBO simulations of MLD and provided a novel approach to evaluate factors associated with MLD pathogenesis, disease progression and new potential therapeutic options.

## 1. Introduction

Metachromatic leukodystrophy (MLD) is an autosomal recessive neurodegenerative metabolic disorder, of the lysosomal storage diseases (LSDs). MLD is one of the most prevalent leukodystrophies (atrophy of the brain’s white matter) and has a prevalence rate of ~1 in 40,000–160,000 worldwide. MLD occurs at a much higher rate in genetically isolated populations [1].

Based on the type of mutation and degree of arylsulfatase A enzyme (ARSA) deficiency, MLD can show a wide spectrum of clinical manifestations that range from cognitive impairment to progressive abnormalities of motor and language function and early death [1,2]. As a secondary complication of MLD, unprocessed fats (i.e., sulfatides) accumulate within lysosomes and lead to lysosome dysfunction and the dysregulation of other lysosomal enzymes, leading to the complete disruption of lysosomal function and the eventual death of the affected cells (i.e., oligodendrocytes and neurons) [3]. As a result of the complex nature of the disease and the lack of understanding of its pathogenesis and progression, there currently are no effective treatments for MLD. However, supportive measures can prolong life for some living with MLD.

### Current and Future Potential for MLD Modeling

It is well known that effective modeling of any disease, including MLD, is crucial to understanding disease pathophysiology, the molecular basis of disease progression and importantly, how to develop effective therapeutic interventions. To date, there are a limited number of MLD models that are based on genetically modified mouse models which do not accurately reflect disease progression in humans [3]. Recently, induced pluripotent stem cell (iPSC) technology was successfully developed to model MLD and this has provided a more personalized or precise tool for studying MLD [4]. Specifically, through the reprogramming of fibroblasts isolated from MLD patients into iPSCs, it is now possible to evaluate the restoration of ARSA activity via gene editing in vitro. Additionally, the differentiation of iPSCs into neuroepithelial progenitor cells, neurons, astrocytes and oligodendrocytes has enabled the study of the molecular and biochemical consequences of ARSA deficiency on different nerve cells. However, advances in iPSC technology continues to be limited by access to rare patient-derived primary somatic cells, high cost and uncertain physiological relevance, because iPSC modeling is based on a single-cell type [5]. Thus, using artificially induced whole-brain organoids (aiWBO) could provide a novel and potentially more physiologically relevant disease modeling approach to MLD. aiWBO simulations have the potential to greatly expand our ability to model MLD and guide future wet lab research and drug repurposing and discovery.

We hypothesize that elucidating the molecular basis of lysosomal dysfunction and the identification of other altered genetic factors using aiWBO simulations of MLD (aiWBO–MLD) will enhance our understanding of the disease and improve our ability to develop more effective treatment strategies. The overarching goal of this research is to guide MLD research by developing novel, more effective treatment strategies for MLD that capitalize on a deepened understanding of the molecular and physiological basis of MLD pathogenesis and disease progression.

## 2. Methods

The DeepNEU (v6.2, 123Genetix, Windsor, ON, Canada) stem cell simulation platform is a literature-validated hybrid deep-machine learning system with elements of fully connected recurrent neural networks (RNN), cognitive maps (CM), support vector machines (SVM) and evolutionary systems (GA). Our DeepNEU platform is built around a large and growing database of relationships between concepts that are derived from gene/protein cell signaling pathways data gleaned from the published literature. The information in the database is stored in a square matrix and matrix values range from −1 for a maximally negative relationship to +1 for a maximally positive relationship. In this system 0 is used to represent an arbitrary baseline value. This fuzzy logic (FL) representation can be converted to a neutrosophic logic (NL) by representing truly unknown relationships with the letter I for indeterminant. An initial state input vector is used to represent the state of the system at time zero. Iterative sparse vector matrix multiplication cycles continue until a new system-wide, steady or final state is achieved. The initial state vector can be modified to represent a wide range of gene-based disorders by turning off or on any combination of gene, protein or phenotypic concepts. Our machine learning platform uses an unsupervised learning approach. In this case, unsupervised learning means there are no data profiles associated with specific outcomes and the system learns relationships between individual concepts rather than a specific number of outcomes. In our approach, unsupervised learning would be closer to clustering and regression than classification. More details about the previous DeepNEU database generation and validation were described in our previous publications [6,7].

In this study we the upgraded DeepNEU database (v6.2) by using the previous data base (v6.1) but adding more than 2000 new genotypic and phenotypic features that were specifically relevant to developing whole-brain organoids and an MLD disease profile. These new features enabled simulation of specific brain organoid cell types, brain regions, the blood–brain barrier (BBB) and microcirculation. Briefly, our previous DeepNEU database (v6.1) contained 4516 proteins or genes and 41,493 nonzero relationships, while the current database (v6.2) contains 4660 proteins or genes and 43,498 nonzero relationships, gene/proteins or phenotypic inputs and outputs.

### 2.1. The Whole-Brain Organoid Simulations, Using NEUBOrg (DeepNEU v6.2) 

The main purpose of this project was to upgrade our recently developed NEUBOrg platform and validated aiWBO simulations using the current version of the DeepNEU (v6.2) machine learning platform. To accomplish this, we used a simulated approach like that published by Yamanaka’s group in 2007 [8] to transform human fibroblasts into what are now known as iPSC. Several important modifications to the original 2007 protocol were required to promote differentiation from artificially induced pluripotent stem cells (aiPSC) to aiWBO. The modified simulated protocol began with the activation of the four Yamanaka (2007) transcription factors, octamer-binding transcription factor 4 (OCT4), cellular myelocytomatosis oncogene (cMYC), kruppel-like factor 4 (KLF4) and sex-determining region Y-box 2 (SOX2). Instead of the iPSC media used by Yamanaka (2007) [8], we substituted a simulated B27 neural media with supplementary zinc and ascorbic acid in the presence of doxycycline, normal levels of ambient oxygen (21%) and increased ambient CO_2_ (5%). The original Yamanaka protocol was carried out under hypoxic conditions and normal levels of CO_2_. The reprogramming of aiPSC to differentiate into aiWBO was conducted in a simulated rotating bioreactor as we have described previously in [7]. All aiWBO simulations were done in triplicate and the simulation protocol is summarized in Table 1. 

The aiWBO simulations constituted multiple relevant neuronal cell types, rostral caudal brain regions, ventral dorsal regions, six horizontal layers of the cerebral cortex and the four layers of the cerebellar cortex. Aiming to reduce system complexity, the spinal cord was not simulated in the current project. The main cell types included were neurons and neural precursor cells (Radial Glial/NSC), astrocytes, oligodendrocytes, oligodendrocyte precursor cells (OPC), interneurons and microglial cells. In addition to the neuronal cell types that were simulated we were also able to simulate endothelial cells and pericytes, which were reported to be important components of the BBB. The forebrain, midbrain and hindbrain were simulated as part of the rostral caudal regions, and the ventral dorsal regions and the ventral forebrain as well as 6 layers of the cerebral cortex were successfully simulated. For more details on the genotypic and phenotypic features that we have used to build the NEUBOrg platform to simulate aiWBO please refer to [9,10,11,12,13,14,15,16,17,18,19,20,21,22,23,24,25,26,27,28,29,30,31,32,33,34,35]. 

### 2.2. The NEUBOrg, aiWBO Simulations Applied to MLD

The validated and unguided NEUBOrg platform was used to simulate a whole-brain organoid affected by ARSA deficiency (MLD). Briefly, the NEUBOrg was upgraded by including previously reported MLD specific phenotypic and genotypic features [4,5,36,37,38,39,40,41,42]. To accomplish the disease modeling simulations (aiWBO–MLD) with NEUBOrg, one key gene concept, ARSA, was locked OFF to simulate *ARSA* gene deletion leading to a complete loss of its function. The simulation processes were otherwise identical to that outlined above for the development of the wild type aiWBO simulations (aiWBO–WT). All aiWBO–MLD and aiWBO–WT simulation experiments were carried out in triplicate. 

To evaluate the performance of the aiWBO–MLD profile with reference to the aiWBO–WT profile, we used genotypic and phenotypic features that were reported to reflect the MLD profile [4,5,36,37,38,39,40,41,42]. Features with negative inputs were used to simulate inhibition of the feature, while features with positive inputs were used to promote the feature. A compilation of the large number and status of inputs that constituted the aiWBO–MLD feature profile is presented in Table 2.

### 2.3. NEUBOrg Platform Statistical Analysis

Consistent with our previous research [7], we used the unbiased binomial test to conduct statistical analysis between the data that were predicted by aiWBO–WT or aiWBO–MLD simulations vs the previously published data. This statistical analysis was selected as it can compensate for prediction bias. To calculate the significance of difference between groups (e.g., aiWBO–WT vs aiWBO–MLD), we used the non-parametric Mann–Whitney U as the data was not normally distributed [43]. 

### 2.4. Drug Target Discovery and Drug Repurposing for Treating MLD

An analysis of potential drug targets was carried out using the unpaired two-tailed T-test to compare the aiWBO–WT and aiWBO–MLD up- and down-regulated genes. In addition to important targets involved in intracellular calcium homeostasis including calpastatin, calpains and voltage-gated calcium channels (VDCC), many kinase pathways were clearly impacted by the ARSA deficiency (all *p* values < 10^−10^). These kinases included multiple members of mitogen-activated protein kinase (MEK), rapidly accelerated fibrosarcoma (RAF), myelocytomatosis protooncogene (MYC), Janus kinase (JAK), protein kinase B (PKB/AKT), Src kinase (SRC), fibroblast growth factor receptors (FGFR), vascular endothelial growth factor receptor (VEGFR), Aurora kinase and Toll-like receptors (TLR). Other affected kinases included the mechanistic target of rapamycin (mTOR), 5’ AMP-activated protein kinase (AMPK), glycogen synthase kinases (GSK3), serine/threonine-protein kinase (SGK), protein kinase C (PKC) and the Ca^2+^/calmodulin-dependent protein kinase (CAMK). Based on this analysis, the decision was made to focus drug repurposing efforts on combinations of multi-kinase inhibitors, more targeted kinase inhibitors and other agents with broad activities. VitD3 was also included in this analysis, because of a marked difference between WT and MLD simulations (*p* value <10^−11^).

Once validated by previously published wet lab results, that the system had not seen before, the drug targets for drug repurposing were identified and are summarized in the results section below. Based on the identified targets, we evaluated a diverse set of targeted and multi-targeted drugs that were approved for other indications or in development. Our approach to identifying effective therapies for MLD was based on the assumptions that (1) while we could not alter the underlying genetic mutation with drugs, we may be able to affect the phenotype, (2) restoring the phenotype to a more normal state could improve disease survival and (3) combinations of targeted and multi-targeted drugs could produce related pathway effects that mitigate the ARSA deficiency and help restore a normal phenotype. To simulate the untreated disease state, we included a placebo. The placebo was created by simply creating a treatment vector composed of all 0s. In other words, no concepts were turned on. 

The impact of treatments was assessed using the previously described MLD disease profile which was equivalent to the placebo or untreated profile. The only modification to the original profile was the exclusion of the ARSA deletion. The ARSA concept was fixed at −1 for all simulations, representing the worst case scenario, namely the deletion of the *ARSA* gene resulting in a complete loss of function. The modified disease profile (N = 9 factors) used here included astrocyte activation, microglial activation, demyelination, dementia, neurodegeneration, peripheral neuropathy, seizures, lysosomal sulfatide and released sulfatide. 

A validated distance measure, the angular cosine distance (ACD), was used to evaluate all treatment effects relative to placebo. The ACD is a statistical metric for representing the distance between two or more real valued vectors. The ACD is based on a widely used similarity measure, the cosine similarity (CS). Once calculated, the CS can be converted to ACD using the formula ACD = arccosine (CS)/Pi. As distance from the reference vector increases the ACD increases towards 1, while decreasing distance from the reference moves the ACD toward 0. Using the ACD it was possible to rank the effect of treatments relative to the placebo. Statistically significant differences between placebo and treatment effects were evaluated using the two-tailed paired t-test and a *p* value < 0.05 was interpreted to indicate that observed differences could not be attributed to chance alone. 

## 3. Results

### 3.1. The NEUBOrg Platform Specification

As described in the method section, the NEUBOrg/DeepNEU database (v6.2) contained a large number of phenotypic and genotypic features, which resulted in the generation of a large amount of data flowing through each node in the RNN. On average, each node in the network initially had >9.3 inputs and >9.3 outputs. The pre-simulation probability of a positive outcome prediction was 0.658 and the pre-test probability of a negative prediction was therefore 0.342. This system bias was used when applying the binomial test to all simulation outcomes to avoid any prediction biases. The spinal cord was not simulated in the current version.

### 3.2. The aiWBO Wild Type Simulations

As we have described previously [7], the aiWBO simulations converged after 31 iterations to a new system-wide steady state without evidence of overtraining after 1000 iterations. The simulation results of aiWBO–WT are outlined below:

1. Simulation of Common Neural Cell Types in aiWBO–WT 

The aiWBO–WT simulations accurately predicted the expression of the common nine neural cell types that are found in human brain organoids. The probability that all (N = 9) cell types were predicted by chance alone is 0.023 (binomial test). These results are summarized in Figure S1A.

2. Simulation of Rostral–Caudal Brain Regions in aiWBO–WT

The aiWBO–WT simulations accurately predicted the expression of all three regions in human brain organoids. These regions are the forebrain, midbrain, and hindbrain. The probability that the expression of all (*N* = 3) regions were predicted by chance alone is 0.285 (binomial test). These results are summarized in Figure S1B.

3. Simulation of Ventral–Dorsal Brain Regions in aiWBO–WT

The aiWBO–WT simulations accurately predicted the expression of all nine ventral (anterior)–dorsal (posterior) regions of human brain organoids. The probability that the expression of all (*N* = 9) regions were predicted by chance alone is 0.023 (binomial test). These results are summarized in Figure S1C.

4. Simulation of Acid-Base status of the aiWBO–WT

The aiWBO–WT simulations accurately predicted the expression of all seven concepts representative of compensated metabolic alkalosis and respiratory acidosis in human brain organoids. The probability that the expression of all (*N* = 7) concepts associated with the expression of a compensated metabolic alkalosis and respiratory acidosis were predicted by chance alone is 0.053 (binomial test). These results are summarized in Figure S1D.

5. Simulations of Cerebral Cortical Layers in aiWBO–WT

The aiWBO–WT simulations accurately predicted the expression of all six cerebral cortical layers in human brain organoids. The probability that the expression of all (*N* = 6) cerebral cortical layers were predicted by chance alone is 0.081 (binomial test). These results are summarized in Figure S2A.

6. Simulation of the Cerebellar Cortical Layers in aiWBO–WT

The aiWBO–WT simulations accurately predicted the expression of all four cerebellar layers cortical in human brain organoids. The probability that the expression of all (*N* = 4) cerebral cortical layers were predicted by chance alone is 0.188 (binomial test). These results are summarized in Figure S2B.

7. Simulation of Microcirculation in aiWBO–WT

The aiWBO–WT simulations accurately predicted the expression of all seven features used to reflect microcirculation in human brain organoids. The probability that the expression of all (*N* = 7) features associated with the expression of a microcirculation were predicted by chance alone is 0.053 (binomial test). These results are summarized in Figure S2C.

8. Blood–Brain Barrier (BBB)

The aiWBO–WT simulations accurately predicted the expression of all seven features that represent BBB in human brain organoids. The probability that the expression of all (*N* = 7) concepts associated with the expression of a BBB were predicted by chance alone is 0.053 (binomial test). These results are summarized in Figure S2D.

## 4. Summary of aiWBO–WT Simulations Profile

aiWBO–WT accurately predicted the expression of 43 elements consistent with wet lab whole-brain organoid profiles, and the presence of seven factors of compensated metabolic alkalosis and respiratory acidosis was also consistent with wet lab findings. The probability that the expression of all (*N* = 50) features were predicted by chance alone is < 0.00000001 (binomial test).

The aiWBO–MLD Simulations

The unsupervised aiWBO–MLD simulations converged after 27 iterations to a new system-wide steady state without evidence of overtraining after 1000 iterations. The simulation results for aiWBO–MLD are outlined below:

1. Simulation of Neural Cell Types in aiWBO–MLD

**Figure 1:**
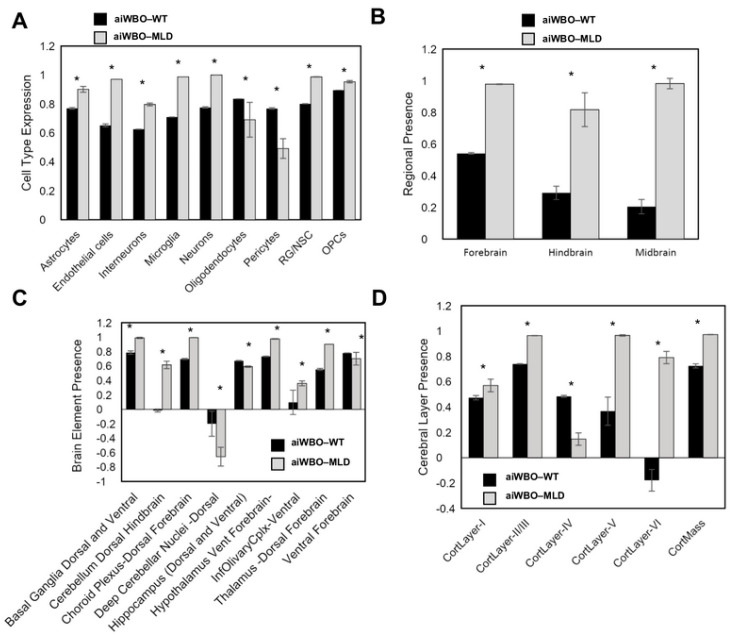
Simulation results of cell types, brain regions and layers in aiWBO–WT vs aiWBO–MLD. (**A**), Comparison of artificially induced whole brain organoid (aiWBO) simulations of wild type (WT) vs Metachromatic Leukodystrophy (MLD) cell types. (**B**), Comparison of brain region concepts in WT vs MLD simulations, (**C**), Comparison of ventral-dorsal concepts in WT vs MLD simulations. (**D**), Comparison of cerebral cortical layers in WT vs MLD simulations. The vertical y-axes represent the semiquantitative levels of concepts that are estimated by DeepNEU relative to an arbitrary base line where 0 = base line, 1 = maximum expression or presence and −1 = minimal expression level or presence. The horizontal x-axes represent the individual aiWBO concepts being simulated. Data represent means of three experiments ± 95% confidence interval. All *p*-values from Mann-Whitney U test. * *p* < 0.01.

The aiWBO–MLD simulations successfully predicted the expression of all nine common neural cell types that are found in human brain organoids. The probability that all (N = 9) cell types were predicted by chance alone is 0.023 (binomial test). A statistical analysis using the Mann–Whitney U test also indicated that there were significant increases in the expression of astrocytes, endothelial cells, interneurons, microglia, OPC, RG/NSC and neurons, while oligodendrocytes and pericytes were significantly decreased in the aiWBO–MLD simulations (all *p* values < 0.01). These results are summarized in Figure 1A.

2. Simulation of Rostral–Caudal Brain Regions in aiWBO–MLD

The aiWBO–MLD simulations successfully predicted the expression of all three rostral–caudal brain regions of human brain organoids, namely, the forebrain, midbrain and hindbrain. The probability that the expression of all (*N* = 3) regions were predicted by chance alone is 0.285 (binomial test). There was a significant (*p* < 0.01) increase in the expression of the forebrain, hindbrain and midbrain in the aiWBO–MLD simulations with reference to aiWBO–WT (Mann–Whitney U test). These results are summarized in Figure 1B.

3. Simulation of Ventral–Dorsal Brain Regions in aiWBO–MLD

The aiWBO–MLD simulations successfully predicted the expression of all nine ventral (anterior)–dorsal (posterior) representative regions that are commonly found in wet lab human brain organoids. The probability that the expression of all (*N* = 9) regions were predicted by chance alone is 0.023 (binomial). There were no significant differences (*p* > 0.05) in the expression of ventral forebrain regions in the aiWBO–WT vs the aiWBO–MLD simulations (Mann–Whitney U test). Several regions were increased in the aiWBO–MLD vs the aiWBO–WT simulations (*p* < 0.01). The increased regions include the basal ganglia (dorsal and ventral), cerebellum (dorsal), choroid plexus (dorsal), hypothalamus (ventral), inferior olivary complex (ventral) and thalamus (dorsal). The decreased regions in the aiWBO–MLD vs the aiWBO–WT simulations (*p* < 0.01) include the deep cerebellar nuclei (dorsal) and the hippocampus (dorsal and ventral). These results are summarized in Figure 1C.

4. Simulation of Cerebral Cortical Layers in aiWBO–MLD

The aiWBO–MLD simulations successfully predicted the expression of all six cerebral cortical layers found in human brain organoids (*p* = 0.081, binomial test). A statistical analysis using the Mann–Whitney U test indicated that there was a significant decrease (*p* < 0.01) in the expression of layer 4 with significant increases observed (*p* < 0.01) in layers 1, 2, 3, 5 and 6. Consistent with these findings, there was also a significant increase in estimated cerebral cortical mass in the MLD simulations. These results are summarized in Figure 1D.

5. Simulation of Cerebellar Cortical Layers in aiWBO–MLD

**Figure 2:**
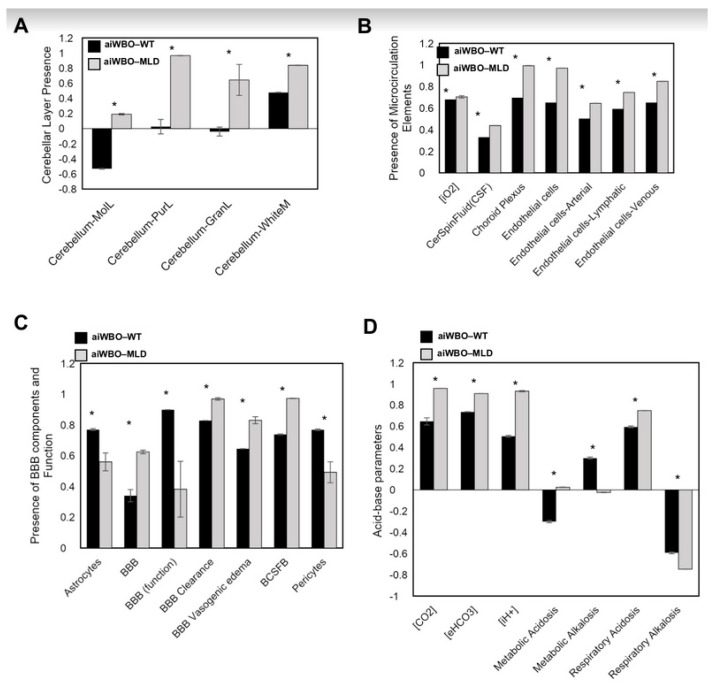
Simulation results for Cerebellar cortical layers microcirculation, BBB and Acid-Base status in aiWBO–WT vs aiWBO–MLD. (**A**), Comparison of cerebellar cortical layers in WT and MLD simulations. (**B**), Comparison of microcirculation elements in WT vs MLF simulations, (**C**), Comparison of blood brain barrier (BBB) concepts in WT vs MLD simulations, (**D**), Comparison of acid base concepts in WT vs MLD simulations. The vertical y-axes represent the semiquantitative levels of concepts that are estimated by DeepNEU relative to an arbitrary base line where 0 = base line, 1 = maximum expression or presence and −1 = minimal expression level or presence. The horizontal x-axes represent the individual aiWBO concepts being simulated. Data represent means of three experiments ± 95% confidence interval. All *p* values from Mann-Whitney U test. * *p* < 0.01.

The aiWBO–MLD simulations successfully predicted the expression of all four cerebellar cortical layers found in human brain organoids. (*p* = 0.188, binomial test). There were significant increases (*p* < 0.01) in the expression of all cerebellar cortical layers in the aiWBO–MLD relative to the aiWBO–WT simulations (Mann–Whitney U test). These results are summarized in Figure 2A.

6. Simulation of Microcirculation in aiWBO–MLD

The aiWBO–MLD simulations correctly predicted the expression of all seven concepts representative of a microcirculation in human brain organoids (*p* = 0.053, binomial test). There were significant increases (*p* < 0.01) in all components of the microcirculation in the aiWBO–MLD relative to the aiWBO–WT simulations. In addition, the intracellular O_2_ concentration was significantly increased (*p* < 0.01) in the aiWBO–MLD simulations consistent with a degree of microcirculation improvement (Mann–Whitney U test). These results are summarized in Figure 2B.

7. Simulation of Blood–Brain Barrier in aiWBO–MLD

The aiWBO–MLD successfully predicted the expression of all six features that represent the BBB profile in human brain organoids (*p* = 0.185, binomial test). Statistical analysis indicated that there were significant decreases (*p* < 0.01) in the astrocyte and pericyte components of the BBB (Mann–Whitney U test). In addition, the function of the BBB was significantly decreased (*p* < 0.01) in the MLD simulations (Mann–Whitney U test). While the expression of the BBB itself was increased (*p* < 0.01) in association with increased clearance (*p* < 0.01), BBB dysfunction appeared to dominate with a significant increase in vasogenic brain edema (*p* < 0.01). Finally, the previously noted increase in choroid plexus was consistent with the significant increase in BCSFB (*p* < 0.01). These results are summarized in Figure 2C.

8. Simulation of Acid-Base Status in aiWBO–MLD 

The expression of all seven features that represent compensated metabolic alkalosis and respiratory acidosis in human brain organoids were predicted successfully and the data are summarized in Figure 2D.

The probability that the expression of all (*N* = 7) concepts associated with the expression of a complex respiratory acidosis were predicted by chance alone is 0.053 (binomial test). There were significant changes (*p* < 0.01, Mann–Whitney U test) in all components of the aiWBO–MLD simulations with reference to aiWBO–WT. Additionally, there was a significant increase in intracellular [H+] concentration in aiWBO–MLD. In summary, these data suggest a less well compensated complex respiratory acidosis with a metabolic component in the aiWBO–MLD simulations. 

9. Disease Modeling Simulations of the aiWBO–MLD

**Figure 3:**
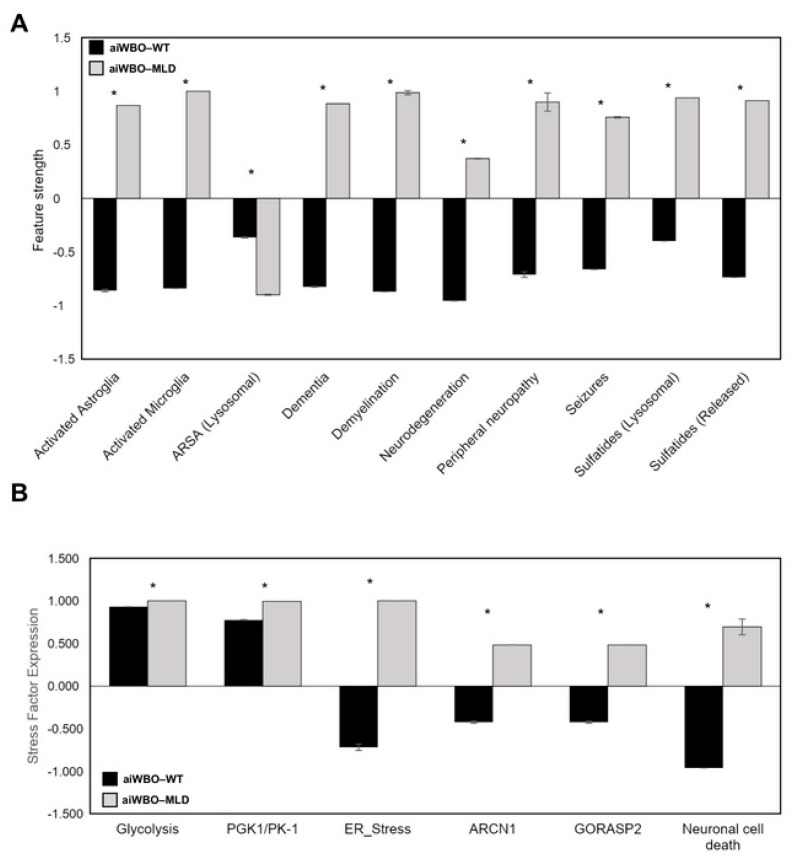
Simulation results of aiWBO–MLD disease profile, cellular stress and neuronal cell death. (**A**), Simulation of MLD features in reference to WT. (**B**), comparative simulation predictions of cellular stress and neuronal cell death in aiWBO–WT vs aiWBO–MLD. The vertical y-axes represent the semiquantitative levels of concepts that are estimated by DeepNEU relative to an arbitrary base line where 0 = base line, 1 = maximum expression or presence and −1 = minimal expression level or presence. The horizontal x-axes represent the individual aiWBO concepts being simulated. Data represent means of three experiments ± 95% confidence interval. All *p* values from Mann-Whitney U test. * *p* < 0.01.

The expression of all 10 features that represent the MLD disease profile were successfully predicted by the aiWBO–MLD simulations. These data are summarized in Figure 3A. The probability that the expression of all (*N* = 10) MLD concepts were predicted by chance alone is 0.015 (binomial test). Statistical analysis indicated that there were significant changes (*p* < 0.01, Mann–Whitney U) in all components of the simulated aiWBO–MLD disease profile with reference to the aiWBO–WT profile. 

10. Cellular Stress and Neuronal Cell Death

The aiWBO–WT and aiWBO–MLD simulations predicted the expression of six markers of cellular stress and neuronal cell death that are commonly expressed in in vitro human brain organoids. A statistical analysis using the Mann–Whitney U test indicated that there were significant changes (*p* < 0.01). These results are summarized in Figure 3B.

## 5. Summary of aiWBO–MLD Disease Profile Simulations

The aiWBO–MLD simulations accurately predicted the expression of 43 elements consistent with a pattern seen in a whole-brain organoid, and seven elements suggesting the expression of a compensated metabolic alkalosis and respiratory acidosis. However, the aiWBO–MLD simulations also produced some variability that is unique to MLD pathogenesis when compared with the aiWBO–WT simulation features. The probability that the expression of all (*N* = 50) concepts were predicted by chance alone is <0.00000001 (binomial test).

### Results of Drug Target Evaluation and Drug Repurposing Efforts

MLD drug targets were identified based on aiWBO–MLD disease profile simulations (Figure 3) and a list of drugs were generated. Specifically, 861 single drug and double drug combinations were generated and evaluated by the final aiWBO–MLD simulations. 41 of the outcomes were for single drugs while the remaining 820 outcomes were the result of applying double drug combinations. All evaluations were carried out in triplicate using starting vectors with the drug and drug combination dose varying between 65 and 85% of the maximum. 

Once the results were ranked by the ACD from the placebo, the top 12 options were selected as an initial group for further study. Interestingly, there were no single drugs in the top 12 options and the double drug options were selected because all had an ACD > 0.972, a *p* value <0.00001 and a probability that at least one of the 12 recommendations would prove true of >0.9999. 

The first-ranked double drug option was predicted to be the multi-kinase inhibitor, regorafenib, plus olaparib, a more targeted kinase inhibitor. Olaparib and lenvatinib were the most frequent members of the top double drug options, each occurring in five of 12 (41.67%) combinations. The multi-kinase inhibitors, regorafenib and sunitinib were elements in three (25%) of the top 12 combinations. Importantly, each of the multi-kinase inhibitors lenvatinib, regorafenib, sunitinib and sorafenib affected at least 10 kinase pathways. Overall, multi-targeted drugs were elements of nine of the 12 top combinations. The highest-ranked single agent was the multi-kinase inhibitor lenvatinib, at 15th in the list. 

All treatment effects were compared to the placebo. A summary of the drug repurposing results and the drugs evaluated in these simulations is given in Table 3 and Table 4.

The data presented in Table 3 were simulated by DeepNEU and represent the semiquantitative levels of concepts that were estimated with and without therapeutic options and estimated relative to an arbitrary base line where 0 = base line, 1 = maximum expression or presence and −1 = minimal expression level or presence. Data represent the mean of three experiments ± 95% confidence. CS refers to cosine similarity and ACD refers to the angular cosine distance metric.

## 6. Discussion

The primary objective of this project was to extend our previous research [7] by upgrading and validating a computer simulation of aiWBO using the latest version of the NEUBOrg/DeepNEU (v6.2) machine learning platform. To that end, we simulated aiWBO–WT and then a rare genetic degenerative neurological disease, namely MLD. Our unsupervised machine learning approach toward the simulation of aiWBO relied on the ability of iPSC to reprogram and differentiate into whole-brain organoids if sufficient time and optimal nutrients and simulation conditions were provided. Our data confirm that the simulations were successful in producing a disease profile like the one seen in the wet lab disease profile (see Figure 3). Additionally, our aiWBO simulations accurately showed all important anatomical and regional aspects of the neonatal brain. Notably, the simulations appeared to have evolved rudimentary elements of a microcirculation and blood–brain barrier. However, both elements were limited by the absence of a functioning cardiovascular system and cerebral circulation. It is important to mention that the current advanced aiWBO simulations were achieved using a cost-effective, highlight-efficient and reproducible method.

We were able to evaluate the acid-base status of aiWBO–WT compared to aiWBO–MLD, because the DeepNEU platform was equipped with the features that enabled the simulation of acid-base status in aiWBO simulations. In summary, we observed that there was a combined metabolic alkalosis and respiratory acidosis in aiWBO–MLD compared with aiWBO–WT. Additionally, there was minimal deviation of the intracellular H+ ion concentration [iH+] from the arbitrary baseline. It is noteworthy that aiWBO–WT and aiWBO–MLD appeared to be adequately oxygenated with levels comparable to the normal ambient oxygen level (21%), while the ambient CO_2_ level was maintained at 5% during the simulation. 

Once the aiWBO simulations were validated against the peer-reviewed literature, the DeepNEU platform was be used to generate aiWBO–MLD simulations, a rare but important demyelinating neurologic disease that primarily affects younger children. A literature-based genotypic and phenotypic 10-feature disease profile was used to assess simulation outputs (see Table 2). Upon comparing the aiWBO simulation predictions with those of the MLD simulations, all simulations correctly reproduced the feature profile seen in in vitro whole-brain organoids. Importantly, the MLD simulations predicted the presence of significantly reduced ARSA levels and the increased lysosomal storage of sulfatides, which are considered important markers for the disease [38]. To our knowledge, this is the first time that whole-brain organoid simulation of MLD has been reported. 

MLD is known to impact at least two important cell types in the central nervous system, namely myelinated neurons and oligodendrocytes, as well as Schwann cells in the peripheral nervous system [44]. While more advanced disease largely results in extensive neuronal loss, the current results would suggest that the MLD simulations may represent an early form of the disease. This conclusion is supported by several other factors. Firstly, the subject age as derived by the simulations at steady state was between birth and 3.5 years of age. Based on current data, this age is widely accepted to be a reasonable period for the emergence of the symptomatic late infantile form of MLD. The simulations indicated that the disease affected primarily oligodendrocytes, the myelin-producing cells of the CNS. Since brain growth, development and regional myelination occur in both wild type and MLD organoids, the increase in the presence of several dorsal and ventral forebrain, midbrain and hindbrain elements may well have been due to a combination of accumulating sulfatides and/or edema which is a common feature of active disease. Alternatively, the neuroinflammation associated with MLD could also have a stimulatory effect on early brain development. These regional increases were supported by an increase in estimated cortical mass. The significant decrease in deep cerebellar nuclei and hypothalamus also suggested more active disease. Active disease is also supported by the predicted disease profile that included the presence neuroinflammation, neurodegeneration, demyelination, dementia, and seizures. Taken together these results are consistent with an early, progressive and symptomatic form of the disease (i.e, late infantile MLD).

We created more anatomically representative three-dimensional (3D) whole-brain simulations by evaluating several features of (1) rostral–caudal regions, (2) ventral–dorsal regions, (3) cerebral cortical and (4) cerebellar cortical layers. Historically, in vitro whole-brain organoids have been relatively poorly organized. The aiWBO–WT and aiWBO–MLD simulations, on the other hand, more accurately reproduced the regional and cortical layer organization seen in the human brain. While these organizational results are encouraging, they are also limited by the paucity of literature-validated markers for some brain regions. Fortunately, the DeepNEU database facilitates the incorporation of new features and this situation will continue to improve as new data becomes available.

Previously, most attempts to produce whole-brain organoids with evidence of a basic microcirculation have met with limited success. Even in the absence of a functioning heart and vascular system, the expression of the elements of a simple cerebral microcirculation is a critical element of a functioning BBB. Several key features of a rudimentary microcirculation present in aiWBO–WT simulations are summarized in Figure S2. In addition, it also appears that the microcirculation may be impaired in the MLD simulations, in keeping with effects seen in patients with MLD.

Notably, features of BBB were also expressed in both aiWBO–WT and aiWBO–MLD simulations. However, significant decreases in the astrocyte and pericyte elements of the BBB were revealed in the MLD simulations relative to aiWBO. In addition, the function of the BBB was also significantly impaired in the MLD simulations, resulting in cerebral edema, which is a common feature of active MLD. While the function of the BBB was not explored in detail in these initial experiments, it has important nutritional, metabolic and pharmacological implications which we continue to evaluate with a focus on drug development. The NEUBOrg platform aiWBO simulations should facilitate efficient identification of drugs and other potential therapeutics which either enter or do not enter the central nervous system.

### 6.1. The Issues of Cellular Stress and Neuron Cell Death

Our data suggest a significant up-regulation of cellular stress as well as neuron cell death markers, as shown in Figure 3B. These data suggest that our aiWBO–MLD simulations accurately reflected phenotypic features associated with MLD in the developing brain.

Validated computer simulations have several important potential advantages [45]. They can be customized, developed and deployed rapidly in a cost-effective manner when compared with wet lab organoid development. We are continuing to address the cellular stress and neuronal cell death that are seen in in vitro and now in simulated whole-brain organoids. It is likely that these significant issues are largely the result of the absence of functioning cardiovascular, pulmonary, and renal systems. It should be faster and easier to optimize organoid cellular stress and neuronal death by modifying the simulated environment as required. The data presented here show the promising potential of aiWBO simulations to overcome the current limitations of developing wet lab brain organoids.

### 6.2. Drug Repurposing for MLD

The aiWBO–MLD simulations were able to identify several potential treatment options that are predicted to have an ameliorating impact on the nine-factor MLD disease profile. Importantly, this is a phenotypic profile in recognition of the fact that drug therapies are unlikely to address the underlying genetic mutation directly. The top candidates appear to impact all nine factors in a beneficial way. All members of an extended group of the 42 top candidates were double drug combinations, except for the multi-kinase inhibitor lenvatinib at 15th on the list. We proposed that double drug combinations would be better candidates for the treatment of MLD based on the growing experience with multi-drug cocktails for treating many other serious diseases. Examples of serious diseases currently treated with approved multi-drug cocktails include many cancers, HIV, tuberculosis, other serious resistant bacterial infections, congestive heart failure, diabetes, hypertension, complex seizure disorders and resistant psychoses to name just a few. It is therefore not surprising that double drug combinations containing multi-targeted drugs dominate the top treatment candidates. We did not consider triple drug combinations in this part of the project because we identified several candidates based on double drug combinations, and cumulative toxicity would likely be increased with three-drug combinations. We attempted to further reduce potential drug-associated cumulative toxicities by only evaluating submaximal doses of all drug candidates. The mean dose for all simulations was 75% of the maximum, although it is anticipated that each drug would be started at a lower dose and titrated up to a maximum of 75% of the recommended maximum doses as tolerated.

As outlined previously, a subset of the top 12 ranked treatment options was selected from a superset of at least 42 options. The selection of this subset of 12 options was based on several factors. Each of the 12 options had a strongly positive ACD > 0.972, indicating that they were almost as far from the placebo/untreated disease profile as possible. Secondly, each of these options was significantly different from the placebo, with two-tailed t-test *p* values < 0.00001. Lastly, while we conclude based on the data that the simulations are likely to be accurate, if we assume a worst-case scenario where simulations are only 50% accurate then the probability that at least 1 of the 12 options is correct is still > 0.9999, and the probability of success increases as the true accuracy of predictions is revealed through initial wet lab testing.

### 6.3. Promises and Pitfalls of the Current aiWBO Simulations

We recognize that a key limitation of this emerging technology is the degree to which the unsupervised NEUBOrg platform can accurately represent currently available whole-brain organoids. These initial data confirm that the platform can recreate a diverse range of previously unseen features and profiles attributed to human brain organoids in the published literature. Currently, any simulation of a biological system like the human brain will be less complex than the organ itself. To complicate this further, our current understanding of the human brain is itself incomplete. Going forward, the key to evaluating the utility of the NEUBOrg platform will be obtaining new data for learning and from ongoing validation. The data in v6.2 represents more than 20% of the human genome compared with ~18% in our earlier versions 5.0 and 6.1. Specifically, we included 6275 new relationships that are relevant to the differentiation of cells into whole-brain. Additionally, the current version of DeepNEU produces more than 9.3 gene/protein/phenotypic inputs and outputs. However, this advanced computer modeling of WBO will greatly benefit from wet lab confirmation. Our ultimate goal for this project is to make our current MLD findings publicly available to researchers, so we can amplify the benefit of using validated machine learning to model rare diseases and fast-track drug discovery.

## 7. Conclusions/Future Directions

As we continue to develop our DeepNEU/NEUBOrg platform, we believe that our ability to model rare human diseases, including MLD, will substantially improve. To date, MLD disease remains largely unexplored due to the limited ability to accurately model MLD, and hence, to study the pathophysiological effect of MLD-causing mutations. We conducted the current study to meet the pressing need for practical new strategies to further our understanding of the mechanism of actions of MLD-causing mutations, and specifically understand how ARSA loss of function mutations affect brain biology and contribute to MLD pathogenesis. We conclude, based on our findings, that our aiWBO–MLD simulations will not only contribute to improving our understanding of MLD pathogenesis, but also can be expanded for the study of other leukodystrophies and neurodegenerative diseases with a genetic basis. Employing machine learning to model human disease will continue to allow us to address previously unanswered questions, and ultimately to identify and implement effective treatments for MLD and other rare diseases that are challenging to model. Finally, the double drug combinations identified in this research as potentially able to restore a more normal phenotype should be confirmed at the earliest possible opportunity.

Optimally, these validated simulation predictions should be combined with wet lab experiments to better understand MLD, the disease and fast-track new potential treatment options. To accomplish this, we are actively seeking research partners and collaborations.

## Figures and Tables

**Table 1:** Unguided aiPSC to artificially induced whole-brain organoids (aiWBO) simulation program.

Simulation Summary	Components (Simulated)
Yamanaka (2007) transcription factors	OCT4, cMYC, KLF4 and SOX2 turned ON
B27 neural media	biotin, amino acids, ascorbate, catalase, cortisol, basic fibroblast growth factor (FGF2/bFGF), glutathione, albumin, insulin, SOD1(Cu/Zn), MnSOD/SOD2, progesterone, retinol/vitamin A, thyroid hormones (T3/T4), transferrin, VitE/Tocopherol, l-carnitine locked ON
Supplements	zinc and doxycycline locked ON
Rotating bioreactor (optimized)	B27 media + [CO_2_] = 5%, [O_2_] = 21%, glucose, temperature = 37 degrees C locked ON and high shear forces locked OFF
Age—evolved by the algorithm	Fetal/neonatal

**Table 2:** Metachromatic leukodystrophy (MLD) feature profile.

MLD Features (*N* = 10)	Genotypic/Phenotypic Feature Inputs
*ARSA* gene **	*N* = 4 (0 negatives and 4 positives)
Astrocyte/astroglial cell activation	*N* = 23 (5 negatives and 18 positives)
Microglial cell activation	*N* = 48 (17 negatives and 31 positives)
Dementia	*N* = 18 (5 negatives and 13 positives)
Demyelination	*N* = 22 (8 negatives and 14 positives)
Neurodegeneration	*N* = 39 (8 negatives and 31 positives)
Sulfatides (lysosomal)	*N* = 4 (1 negative and 3 positives)
Sulfatides (released)	*N* = 3 (0 negatives and 3 positives)
Seizures	*N* = 14 (5 negatives and 9 positives)
Peripheral neuropathy	*N* = 5 (0 negative and 5 positives)

** *ARSA* gene was set to −1 and locked to simulate the *ARSA* gene deletion in MLD.

**Table 3:** Effect of top double drug combinations on aiWBO–MLD.

Therapeutic Option	Activated Astroglia	Activated Microglia	Dementia	Demyelination	Neurodegeneration	Peripheral Neuropathy	Seizures	Sulfatides (Lysosomal)	Sulfatides (Released)	CS	ACD	*t*–Test *p* Value
Placebo	0.9	0.8	0.7	0.9	0.4	0.9	0.7	0.9	0.9	1.0	0.0	1.0
Regorafenib + Olaparib	−0.9	−0.9	−0.6	−1.0	−0.4	−0.8	−0.7	−0.8	−0.9	−1.0	1.0	<0.00001
Pembrolizumab + Lenvatinib	−0.9	−0.9	−0.7	−0.9	−0.3	−0.8	−0.7	−0.8	−0.9	−1.0	1.0	<0.00001
Sunitinib+Lenvatinib	−0.8	−0.8	−0.6	−0.9	−0.3	−0.8	−0.7	−0.9	−0.9	−1.0	1.0	<0.00001
Lenvatinib + Capmatinib	−0.8	−0.9	−0.7	−1.0	−0.3	−0.8	−0.7	−0.9	−0.9	−1.0	1.0	<0.00001
Rapamycin + Lenvatinib	−0.8	−0.9	−0.6	−1.0	−0.3	−0.8	−0.7	−0.9	−0.9	−1.0	1.0	<0.00001
Regorafenib + Lenvatinib	−0.9	−0.9	−0.7	−1.0	−0.3	−0.8	−0.7	−0.9	−0.9	−1.0	1.0	<0.00001
Regorafenib + Calpain Inhibitor	−0.9	−0.9	−0.6	−1.0	−0.3	−0.8	−0.7	−0.8	−0.9	−1.0	1.0	<0.00001
Sunitinib + Nutlin3	−0.8	−0.8	−0.6	−0.9	−0.3	−0.8	−0.6	−0.8	−0.8	−1.0	1.0	<0.00001
Sunitinib + Olaparib	−0.8	−0.9	−0.7	−1.0	−0.4	−0.8	−0.7	−0.9	−0.9	−1.0	1.0	<0.00001
Olaparib + Abemaciclib	−0.8	−0.9	−0.6	−1.0	−0.4	−0.8	−0.6	−0.8	−0.8	−1.0	1.0	<0.00001
Palbociclib + Olaparib	−0.8	−0.9	−0.6	−1.0	−0.4	−0.8	−0.6	−0.8	−0.8	−1.0	1.0	<0.00001
Ribociclib + Olaparib	−0.8	−0.9	−0.6	−1.0	−0.4	−0.8	−0.6	−0.8	−0.8	−1.0	1.0	<0.00001

The data presented in Table 3 were simulated by DeepNEU and represent the semiquantitative levels of concepts that were estimated with and without therapeutic options and estimated relative to an arbitrary base line where 0 = base line, 1 = maximum expression or presence and −1 = minimal expression level or presence. Data represent the mean of three experiments ± 95% confidence. CS refers to cosine similarity and ACD refers to the angular cosine distance metric.

**Table 4:** Summary of the drugs included in this repurposing project.

Drug Class	Class Examples
Ca++ Homeostasis is Agents, *N* = 3	Calcium Channel Blocker (VDCC), Calpain Inhibitor, Calpastatin Agonist
Check point Inhibitor, *N* = 2	Ipilimumab, Pembrolizumab
Chemotherapy agents, *N* = 5	Cisplatin, Dichloroacetate, Doxorubicin, Gemcitabine, Taxol
Multitargeted agents, *N* = 14	Afatinib, Alectinib, Brigatinib, Cabozantinib, Capmatinib, Crizotinib, Imatinib
Targeted agents, *N* = 16	Bortezomib, Nutlin3, Olaparib, Rapamycin, Tamoxifen, Vitamin D3, Anakinra. Jak2 inhibitor, Rituximab, Tocilizumab, Abemaciclib, Bevacizumab, Cetuximab, Palbociclib, Enhertu, Ribociclib
Other, *N* = 1	Placebo

## Supplementary Materials

The following are available online at https://www.mdpi.com/article/10.3390/biomedicines9040440/s1.
