# Supplementary material for: Artificially Induced Pluripotent Stem Cell-Derived Whole-Brain Organoid for Modelling the Pathophysiology of Metachromatic Leukodystrophy and Drug Repurposing
Source: Biomedicines. 2021 Apr 20;9(4):440. doi: 10.3390/biomedicines9040440 (PMC8073899; doi:10.3390/biomedicines9040440)
Supplement: Supplementary file 1 [file biomedicines-09-00440-s001.zip › biomedicines-1153253-supplementary.pdf]

Supplemental figures

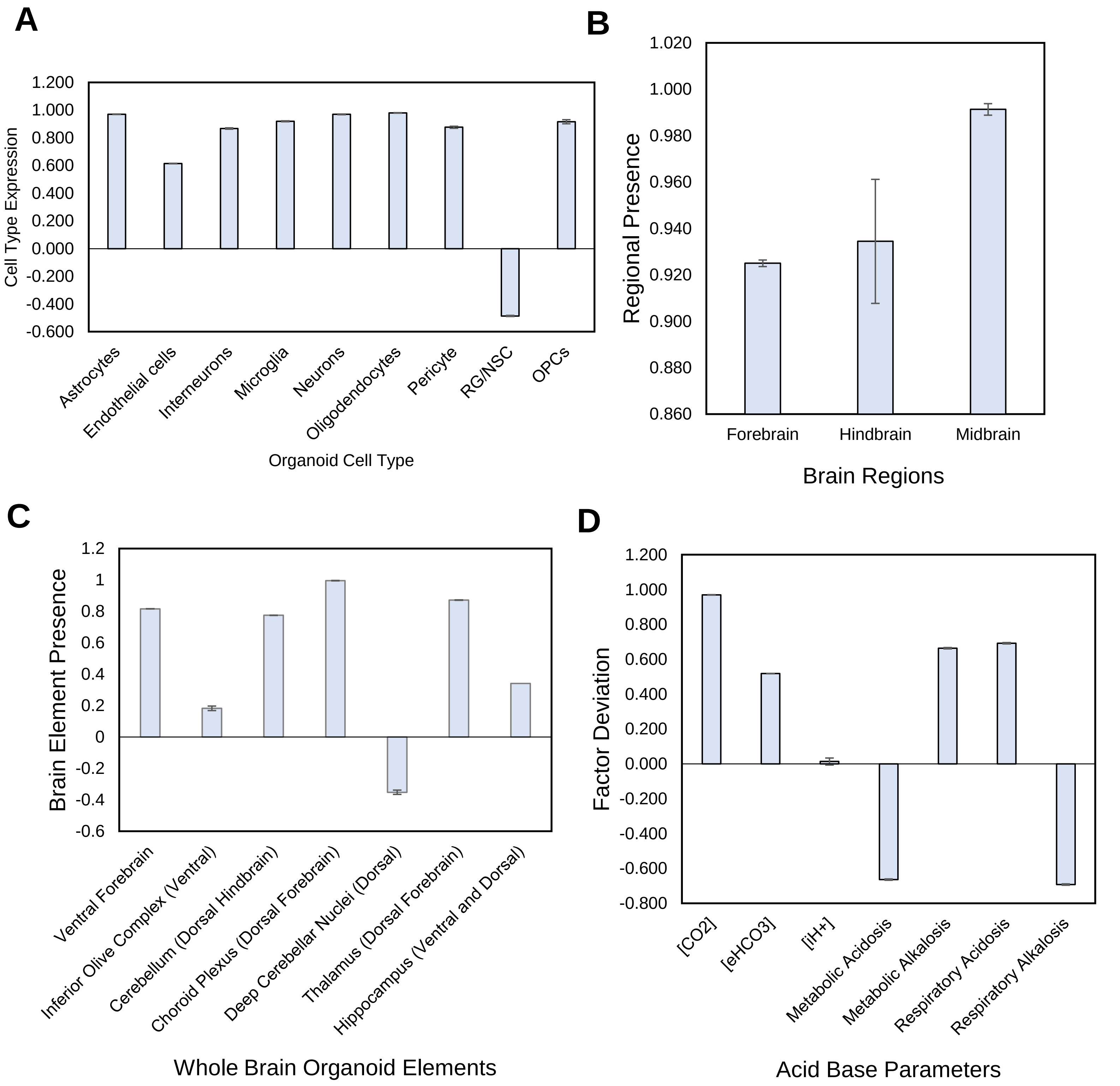

FIGURE S1: Siimulations of differentiated WT aiWBO cell types, regions, elements and acid base status. A, Expression of basic aiWBO cell types based on genotypic markers. B, Presence of the three aiWBO brain regions based on genotypic markers. C, Presence of the ventral and dorsal aiWBO brain elements based on genotypic markers. D, The acid base status of the aiWBO based on phenotypic factors. The vertical y-axes represent the semiquantitative levels of concepts that are estimated by DeepNEU relative to an arbitrary base line where 0 = base line, 1 = maximum expression or presence and -1 = minimal expression level or presence. The horizontal x-axes represent the individual aiWBO concepts being simulated. Data represent mean of three experiments ± 95% confidence interval.

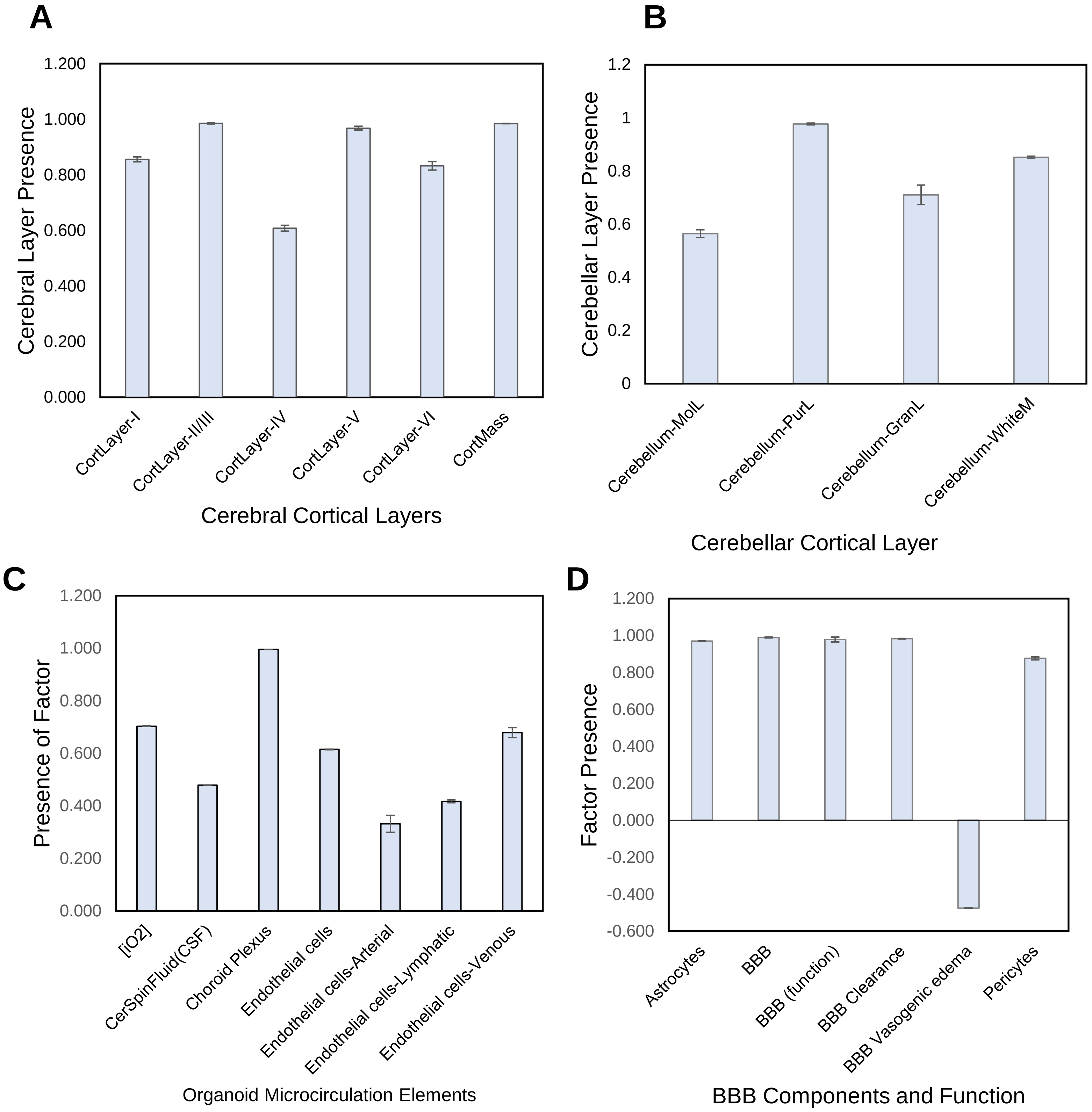

FIGURE S2: Simulations of cerebral cortical layers, cerebellar cortical layers, microcirculation and BBB of WT aiWBO. A, Presence of the six aiWBO cerebral cortical layers based on genotypic markers. An estimate of cortical mass is derived by combining the six cortical layers. B, Presence of the four cerebellar layers based on genotypic markers. C, Presence of aiWBO microcirculation elements based on phenotypic markers. D, The presence of aiWBO BBB elements and function based on phenotypic factors. The vertical y-axes represent the semiquantitative levels of concepts that are estimated by DeepNEU relative to an arbitrary base line where 0 = base line, 1 = maximum expression or presence and -1 = minimal expression level or presence. The horizontal x-axes represent the individual aiWBO concepts being simulated. Data represent mean of three experiments ± 95% confidence interval.
